# Clinical development and marketing application review times for novel orphan-designated drugs

**DOI:** 10.3389/fmed.2024.1404922

**Published:** 2024-06-05

**Authors:** Ebru Demirci, Jennifer Knicley, Lori Fiorentino

**Affiliations:** Global Regulatory Affairs, Pharming Group N.V., Leiden, Netherlands

**Keywords:** orphan-designated drugs, novel drugs, orphan drugs, clinical development, clinical development time, marketing application review time, European Medicines Agency (EMA), US Food and Drug Administration (FDA)

## Abstract

Development of an orphan-designated drug has been more challenging and financially less attractive than that of other drugs due to low prevalence of the condition, poorly defined biomarkers and lack of experience of healthcare providers in diagnosing and treating the condition. Guidance and incentives in some countries support the sponsors in developing orphan-designated drugs despite the challenges. Expedited regulatory programs as offered by the United States (US) Food and Drug Administration (FDA) and the European Medicines Agency (EMA) support the development of drugs, provide shorter marketing application review times or provide preliminary approval. In this study, we analyze marketing application review times in the US and in the European Union (EU) and clinical development times for novel, i.e., containing new molecular entity, orphan-designated drugs that were approved in the US between 1 June 2020 and 31 May 2023, and their correlation with expedited regulatory programs. Seventy-three marketing applications for novel orphan-designated drugs were approved by the FDA, and 39 also received a positive opinion from the EMA. The marketing application review time by the FDA for the 73 novel orphan-designated drugs approved in the US was 244 days (*n* = 73, median), and the marketing application review time by the EMA for the 39 drugs that were also approved in the EU was 353 days (*n* = 39, median). The typical clinical development time for a novel orphan-designated drug was 7.2 years (*n* = 72).

## 1 Introduction

Development of an orphan-designated drug has been more challenging and financially less attractive than that of other drugs due to low prevalence of the condition, difficulty in applying randomization and controls in trial design, geographical dispersion of the patients, poorly defined biomarkers and lack of experience of healthcare providers in diagnosing and treating the condition (1, 2). Regulatory agencies across the globe employ different categories of qualification criteria for a drug to be designated as orphan, including prevalence of the condition (affects fewer than 200,000 persons in the United States (US), or not more than 5 in 10,000 in the European Union (EU)), severity of the disease or availability of adequate treatments (3, 4). Guidance and incentives in some countries, such as priority review vouchers, fee reductions, protocol assistance, and extension of market exclusivity^1^, support the sponsors in developing orphan-designated drugs despite the challenges (1). In 2022, 54% of new drugs approved by the US Food and Drug Administration (FDA)’s Center for Drug Evaluation and Research (CDER) had orphan designation (5).

In addition, not specific to but applicable also for orphan-designated drugs, expedited regulatory programs as offered by the FDA and the European Medicines Agency (EMA), in the US and the EU, respectively, support the development of drugs (e.g., breakthrough therapy designation (BTD) and fast track designation (FTD) in the US, and priority medicines scheme (PRIME) in the EU), provide shorter marketing application review times (e.g., priority review designation in the US, and accelerated assessment in the EU) or provide preliminary approval (e.g., accelerated approval in the US, and exceptional circumstances and conditional marketing authorization in the EU) (6).

Here, we analyze marketing application review times in the US and in the EU and clinical development times for novel, i.e., containing new molecular entity, orphan-designated drugs that were approved in the US between 1 June 2020 and 31 May 2023, and their correlation with expedited regulatory programs. The results of the analysis demonstrate the shortest paths of orphan-designated drug development and provide the most recent real-world regulatory strategy examples to the sponsors of orphan-designated drugs.

## 2 Method

Data on marketing application and approval of drugs in the US, i.e., a list of New Drug Applications (NDAs), Biologics License Applications (BLAs), biosimilars and supplements approved from 1997 by the US FDA, and in the EU, i.e., a list of all centralized products approved since their first European public assessment report (EPAR), and products withdrawn and suspended since 1 March 2012, were obtained from the Clarivate Cortellis Regulatory Intelligence^2^ solution on 31 May 2023.

To construct the dataset using the data on marketing application and approval of drugs in the US, only the drugs marketing application of which was reviewed by CDER and approved between 1 June 2020 and 31 May 2023, with orphan-designation, with active substance status “new active substance,” and with application/submission type “BLA” or “NDA” were included. Drugs with FDA chemical type “new active ingredient” were excluded in order to include only new molecular entities. The active ingredient pitolisant was excluded since it was approved in 2019. The indications for active ingredients asciminib and umbralisib were adjusted based on information available at the Drugs@FDA database. The dataset consisted of 73 novel orphan-designated drugs that were approved by the FDA’s CDER between 1 June 2020 and 31 May 2023. Thirty nine of these drugs were also approved in the EU, however only 26 of these drugs were orphan designated in the EU. Of the 39 drugs approved in the EU, fexinidazole was included, which obtained positive scientific opinion on medicine for use outside of the EU in accordance with Article 58 Regulation (EC) No 726/2004. The registration status, submission date and the Committee for Medicinal Products for Human Use (CHMP) opinion date for active ingredient fexinidazole and the active substance status for active ingredients avalglucosidase alfa and melphalan flufenamide were adjusted based on information in the corresponding EPARs.

Marketing application review times by the FDA and the EMA were calculated as the days elapsed from the marketing application submission date to the date of approval in the US, and the days elapsed from the marketing application submission date to the date of CHMP opinion in the EU.

Data on clinical development, i.e., clinical trial results, were obtained from the Clarivate Cortellis Clinical Trials Intelligence^3^ solution on 13 June 2023. The dataset related to 72 novel orphan-designated drugs (asciminib counted once) was constructed with the following inclusion criteria:

I. The active ingredient is one of the primary interventions. If the active ingredient is named differently in the US and in the EU, any of its names is one of the primary interventions. For combination products, the combination of all active substances in the product is one of the primary interventions.

For the following cases where there are no trials including the active ingredient(s) as one of the primary interventions:

- For combination product atoltivimab, maftivimab, and odesivimab, REGN-EB3 (code name of the product based on the information available at Drugs@FDA) is one of the primary interventions.
- For the active ingredient copper Cu 64 dotatate, DETECTNET is one of the primary interventions.

II. The start date and/or end date is earlier than the marketing application submission date in the US or in the EU, whichever is earlier. In the case where the start date and/or end date is not available, these dates were retrieved from clinicaltrials.gov using the identifier (NCT number).

For the clinical trial NCT02218047, start date was adjusted based on information available at clinicaltrials.gov.

The dataset contained 1,050 trials at different clinical development phases, namely 396 Phase 1 trials (including Phase 1, Phase 1a, Phase 1, Phase 1/2 (where enrollment count is under 100 or not specified) and Phase 0), 480 Phase 2 trials (including Phase 2, Phase 2a, Phase 2b, Phase 1/2 (where enrollment count is 100 or over), Phase 2/3 (where enrollment count is under 300 or not specified)), 149 Phase 3 trials (including Phase 3, Phase 3b, Phase 3a, Phase 2/3 (where enrollment count is 300 or over)), 10 Phase 4 trials and 15 trials with no assigned phase (i.e., Phase Not Applicable).

Clinical development time was calculated as the number of days converted to years that elapsed from the earliest clinical trial start date to the marketing application submission date in the US or in the EU, whichever is earlier.

Clinical development time for each clinical development phase was calculated as the number of days converted to years that elapsed from the earliest clinical trial start date for the corresponding phase to the earliest of the marketing application submission date in the US or in the EU, whichever is earlier, or the latest clinical trial end date for the corresponding phase.

## 3 Results

### 3.1 Marketing application review times for novel orphan-designated drugs

Seventy-three marketing applications for novel orphan-designated drugs were approved by the FDA’s CDER between 1 June 2020 and 31 May 2023. Out of the 73 novel orphan-designated drugs approved in the US, 39 also received a positive opinion from the CHMP of the EMA, with 26 of these drugs also being orphan designated in the EU. Additionally, 36 of the 39 drugs approved in the EU had new active substance status. Asciminib was approved with two different indications in the US, and only one of these indications was approved in the EU.

The marketing application review time by the FDA for the 73 novel orphan-designated drugs approved in the US, defined as the days elapsed from the marketing application submission date to the date of approval in the US, was 244 days (*n* = 73, median).

The marketing application review time by the EMA for the 39 drugs that were also approved in the EU, defined as the days elapsed from the marketing application submission date to the date of CHMP opinion in the EU, was 353 days (*n* = 39, median).

**Figure 1:**
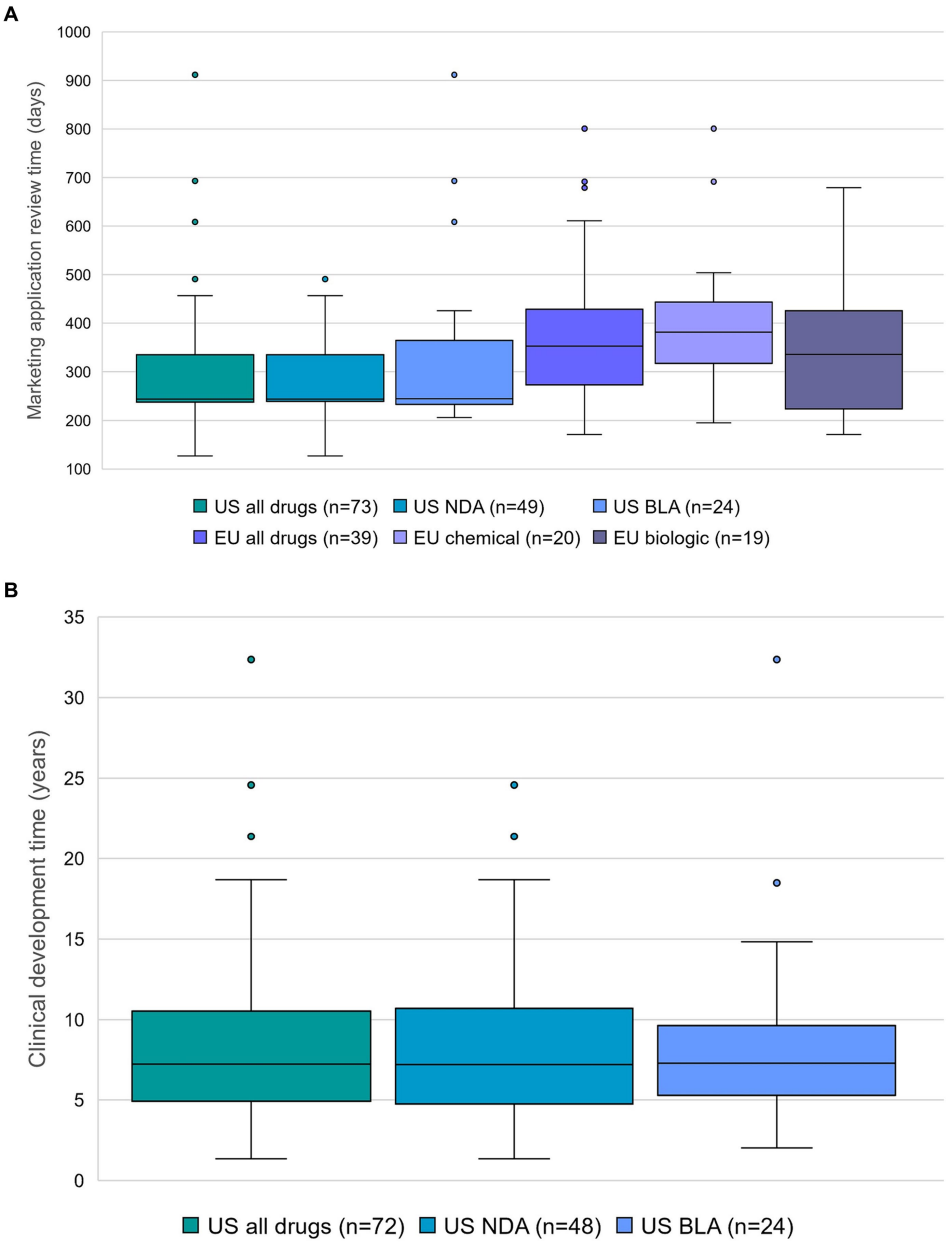
**(A)** Marketing application review time, and application type in the US (new drug application (NDA) and biologics license application (BLA)) or type of product in the EU. Outliers are represented as dots, except for terlipressin (4,884 days in categories US all drugs and US NDA). Data source: Cortellis Regulatory Intelligence. **(B)** Clinical development time and application type in the US. Center lines show the medians. Whiskers extend to minimum and maximum values. Outliers are represented as dots. Number of data points is displayed for each category. Data source: Cortellis Clinical Trials Intelligence.

Application type (NDA or BLA) did not have a notable impact on the marketing application review time by the FDA (244 days (*n* = 49, median) for NDA and 245 days (*n* = 24, median) for BLA), whereas the type of product (chemical or biologic) did have an impact on the marketing application review time by the EMA (382 days (*n* = 20, median) for chemical and 336 days (*n* = 19, median) for biologic) (Figure 1A).

Sixty six of 73 novel orphan-designated drugs approved in the US (90%) and 19 of 39 drugs that were also approved in the EU (49%) were granted expedited regulatory programs in the US and in the EU, respectively. These drugs typically had shorter marketing application review times than the drugs that were not subject to expedited regulatory program (455 days (*n* = 7, median) in the US and 399 days (*n* = 20, median) in the EU). Specifically, the review time of the marketing applications of drugs that were granted priority review designation in the US and accelerated assessment in the EU typically reflected the shortest review time in both regions (Table 1).

**Table 1:** Marketing application review time and expedited regulatory programs.

Expedited regulatory program			US Marketing application review time			EU Marketing application review time		
To support the development of drugs^a^	To provide shorter marketing application review times^b^	To provide preliminary approval^c^	Number of drugs	Median (days)	Range (days)	Number of drugs	Median (days)	Range (days)
No	No	No	7	455	289–912	20	399	286–692
No	No	Yes	0	n/a	n/a	11	372	171–801
No	Yes	No	6	241	201–491	1	216	n/a
No	Yes	Yes	6	244	219–456	2	210	195–224
Yes	No	No	5	365	335–425	1	328	n/a
Yes	No	Yes	1	363	n/a	0	n/a	n/a
Yes	Yes	No	28	245	206–4,884	3	225	198–219
Yes	Yes	Yes	20	236	127–335	1	218	n/a

n/a, not applicable.

a Breakthrough therapy designation (BTD) or fast track designation (FTD) in the US, PRIME in the EU.

b Priority review designation in the US, Accelerated assessment in the EU.

c Accelerated approval in the US, Exceptional circumstances/Conditional marketing authorization in the EU.

### 3.2 Clinical development times for novel orphan-designated drugs

The result of the current analysis shows that the typical clinical development time for a novel orphan-designated drug approved between 1 June 2020 and 31 May 2023 in the US, calculated as the number of days converted to years that elapsed from the earliest clinical trial start date to the marketing application submission date in the US or in the EU, whichever is earlier, was 7.2 years (*n* = 72). Application type (NDA or BLA) did not have a notable impact on the clinical development time (7.2 years (*n* = 48, median) for NDA and 7.3 years (*n* = 24, median) for BLA) (Figure 1B).

**Figure 2:**
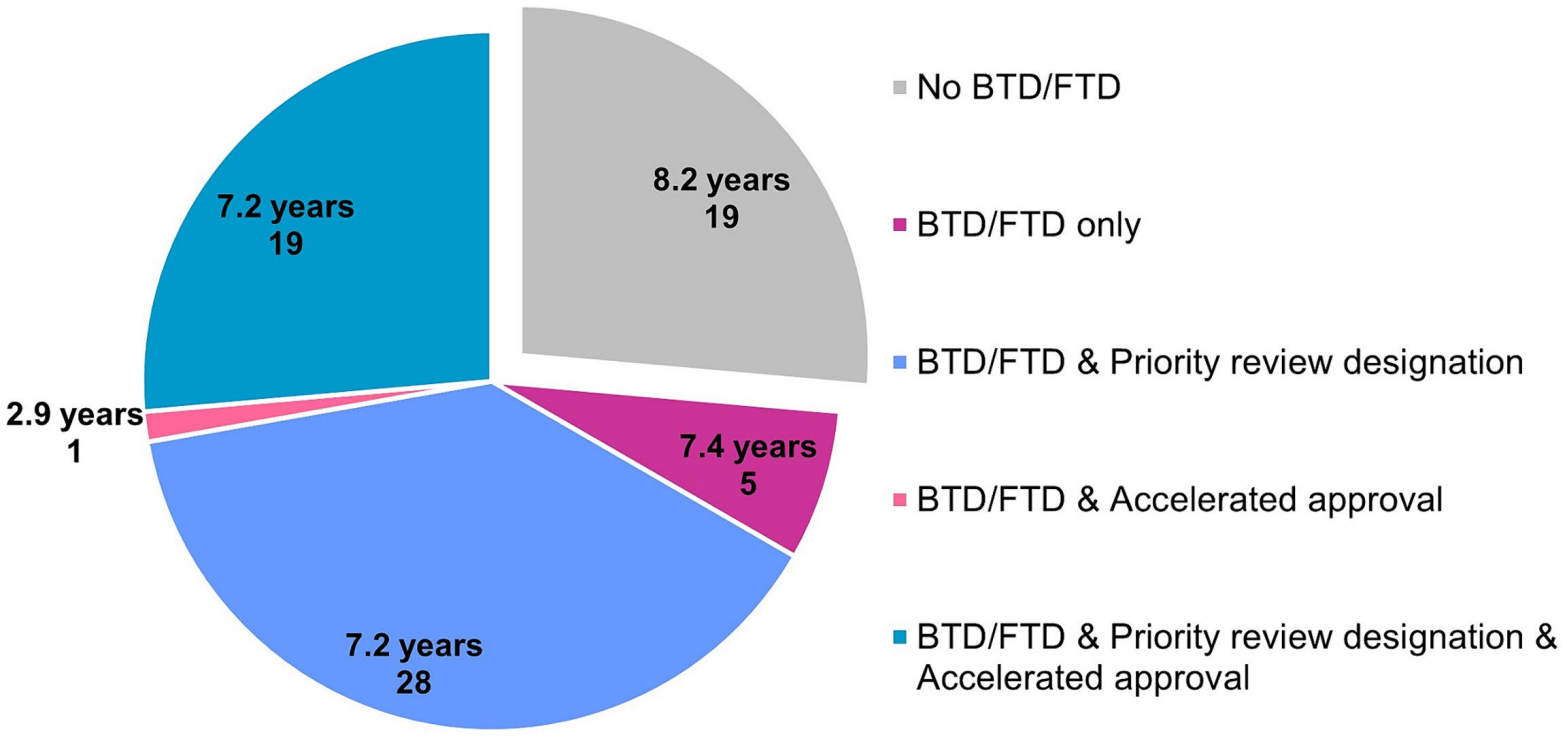
Clinical development time (median) and expedited regulatory programs provided by the FDA for 72 drugs that went or did not go through breakthrough therapy designation (BTD) or fast track designation (FTD). Number of data points is displayed for each category. Data source: Cortellis Clinical Trials Intelligence.

Fifty three of 73 novel orphan-designated drugs (74%) benefited from expedited regulatory programs BTD and/or FTD, and their clinical development time was 7.2 years (*n* = 53, median). Specifically, five utilized only BTD and/or FTD, 28 utilized BTD and/or FTD and priority review designation, and 19 utilized BTD and/or FTD, priority review designation and accelerated approval. The typical clinical development time for these drugs was 7.4 years (*n* = 5), 7.2 years (*n* = 28), and 7.2 years (*n* = 19), respectively. One drug (adagrasib) went through BTD, FTD and accelerated approval, and its clinical development lasted 2.9 years. On the other hand, the clinical development time of 19 drugs (26%) that did not go through BTD or FTD was 8.2 years (*n* = 19, median), indicating the positive impact of having BTD or FTD in the US on shortening the clinical development time (Figure 2). Only 5 drugs utilized PRIME in the EU, together with BTD and/or FTD and priority review in the US (olipudase alfa, setmelanotide, lumasiran and risdiplam) or with BTD and/or FTD, priority review designation and accelerated approval in the US (belantamab mafodotin), with varying clinical development times between 3.9 years and 14.8 years.

A typical novel orphan-designated drug was studied in three Phase 1, four Phase 2, and two Phase 3 clinical trials before its marketing application is submitted. Phase 1, 2 and 3 trials in total for each phase typically lasted 5.4 years, 5.4 years and 2.7 years, typically involving 40, 92 and 284 planned or actual subjects per trial (average), respectively (Table 2 and Supplementary Figure S1).

**Table 2:** Number of trials, clinical development time, and average enrollment per trial for novel orphan-designated drugs at each clinical development phase.

Phase	Number of trials	Time per phase (years)	Average enrollment count per trial
Phase 1	3 (Range: 0–89)	5.4 (Range: 0.2–24.2)	40 (Range: 8–422)
Phase 2	4 (Range: 0–113)	5.4 (Range: 0.4–32.4)	92 (Range: 3–822)
Phase 3	2 (Range: 0–17)	2.7 (Range: 0.0–18.7)	284 (Range: 16–945)

## 4 Discussion

The typical marketing application review time by the FDA for a novel orphan-designated drug approved in the US between 1 June 2020 and 31 May 2023 was 244 days (*n* = 73, median). This result shows that the typical marketing application review time by the FDA for a novel orphan-designated drug has shortened compared to that for a novel orphan-designated drug approved between 2010 and 2020 (360 days, *n* = 40% of 405) in the US, as reported by Brown et al. (7).

The typical marketing application review time by the EMA for the drugs that were also approved in the EU was 353 days (*n* = 39, median), 109 days longer than the typical marketing application review time by the FDA. This result is in line with the previously published studies showing that the marketing application review time in the EU is longer than that in the US, for orphan designated as well as for other drugs (8–11).

Ninety % of novel orphan-designated drugs approved in the US and 49% of drugs that were also approved in the EU were granted expedited regulatory programs in the US and in the EU, respectively, demonstrating that use of expedited regulatory programs is much less frequent in the EU than in the US, confirming the findings of previous studies (10, 12, 13). Drugs that were granted priority review designation in the US and accelerated assessment in the EU typically had the shortest marketing application review time. A notable impact of the type of product (chemical or biologic) was observed on the marketing application review time by the EMA.

The typical clinical development time for a novel orphan-designated drug approved between 1 June 2020 and 31 May 2023 in the US was 7.2 years (*n* = 72), shorter than 10.6 years (time between the initiation of first-in-human clinical study and regulatory marketing authorization) as reported for orphan-designated drugs approved by the FDA between 2010 and 2020 (7).

Seventy four % of novel orphan-designated drugs benefited from expedited regulatory programs BTD and/or FTD, and their clinical development time was 7.2 years (*n* = 53, median). The clinical development time of 26% of the drugs that did not go through BTD or FTD was 8.2 years (*n* = 19, median), indicating the positive impact of having BTD or FTD in the US on shortening the clinical development time.

The dataset consisting of 1,050 trials might not include all clinical trials on the active ingredients, including the actual first-in-human studies which are ideally regarded as the start of clinical development for drugs. In addition, the dataset might contain clinical trials not related to the approved indication of the corresponding active ingredient. Moreover, additional confirmatory clinical development after marketing application submission, which is mandatory for the drugs approved via accelerated approval in the US or conditional marketing authorization in the EU, was not taken into account in this study.

## Data availability statement

The data analyzed in this study is subject to the following licenses/restrictions: Access is restricted to protect confidential or proprietary information. Requests to access these datasets should be directed to https://clarivate.com/products/biopharma/regulatory-compliance/regulatory-intelligence-solutions/ or https://clarivate.com/products/biopharma/research-development/clinical-trials-intelligence-analytics/.

## Author contributions

ED: Conceptualization, Formal analysis, Investigation, Visualization, Writing – original draft, Writing – review & editing. JK: Validation, Writing – review & editing. LF: Writing – review & editing.

## Footnotes

^1^Orphan designation: Overview. https://www.ema.europa.eu/en/human-regulatory-overview/orphan-designation-overview#after-orphan-designation-11926.

^2^Cortellis Regulatory Intelligence. https://clarivate.com/products/biopharma/regulatory-compliance/regulatory-intelligence-solutions/.

^3^Cortellis Clinical Trials Intelligence. https://clarivate.com/products/biopharma/research-development/clinical-trials-intelligence-analytics/.

## References

[1] Korth-Bradley JM. Regulatory framework for drug development in rare diseases. J Clin Pharmacol. (2022) 62:S15–26. doi: 10.1002/jcph.2171 36461739

[2] Giannuzzi V Conte R Landi A Ottomano SA Bonifazi D Baiardi P. Orphan medicinal products in Europe and United States to cover needs of patients with rare diseases: an increased common effort is to be foreseen. Orphanet J Rare Dis. (2017) 12:64. doi: 10.1186/s13023-017-0617-1, PMID: 28372595 PMC5376695

[3] U.S. Department of Health and Human Services, Food and Drug Administration, Office of Orphan Products development (OOPD). Clarification of Orphan Designation of Drugs and Biologics for Pediatric Subpopulations of Common Diseases: Guidance for Industry. (2018)

[4] Commission, REGULATION (EC) no 847/2000 of 27 April 2000, (2000)

[5] Mullard A. 2022 FDA approvals. Nat Rev Drug Discov. (2023) 22:83–8. doi: 10.1038/d41573-023-00001-3 36596858

[6] Demirci E Omes-Smit G Zwiers A. Clinical development time is shorter for new anticancer drugs approved via accelerated approval in the US or via conditional approval in the EU. Clin Transl Sci. (2023) 16:1127–33. doi: 10.1111/cts.13519 37013379 PMC10339697

[7] Brown DG Wobst HJ Kapoor A Kenna LA Southall N. Clinical development times for innovative drugs. Nat Rev Drug Discov. (2022) 21:793–4. doi: 10.1038/d41573-021-00190-9, PMID: 34759309 PMC9869766

[8] Joppi R Bertele V Vannini T Garattini S Banzi R. Food and drug Administration vs European medicines agency: review times and clinical evidence on novel drugs at the time of approval. Br J Clin Pharmacol. (2020) 86:170–4. doi: 10.1111/bcp.14130, PMID: 31657044 PMC6983504

[9] Uyl-de Groot CA Heine R Krol M Verweij J. Unequal access to newly registered cancer drugs leads to potential loss of life-years in Europe. Cancer. (2020) 12:2313. doi: 10.3390/cancers12082313, PMID: 32824444 PMC7464890

[10] Da Costa GF Demirci E Zwiers A. A detailed analysis of expedited regulatory review time of marketing authorization applications for new anticancer drugs in the US and EU. Clin Transl Sci. (2022) 15:1959–67. doi: 10.1111/cts.13308 35561071 PMC9372420

[11] Centre for Innovation in Regulatory Science. R&D Briefing 88: New drug approvals in six major authorities 2013–2022: Focus on orphan designation and facilitated regulatory pathways. London, UK: Centre for Innovation in Regulatory Science (CIRS) (2023).

[12] Leo CP Hentschel B Szucs TD Leo C. FDA and EMA approvals of new breast cancer drugs—a comparative regulatory analysis. Cancer. (2020) 12:437. doi: 10.3390/cancers12020437, PMID: 32069837 PMC7072445

[13] Hwang TJ Ross JS Vokinger NN Kesselheim AS. Association between FDA and EMA expedited approval programs and therapeutic value of new medicines: retrospective cohort study. BMJ. (2020) 371:m3434. doi: 10.1136/bmj.m3434 33028575 PMC7537471

