# Supplementary material for: Clinical development and marketing application review times for novel orphan-designated drugs
Source: Front Med (Lausanne). 2024 Jun 5;11:1404922. doi: 10.3389/fmed.2024.1404922 (PMC11188924; doi:10.3389/fmed.2024.1404922)
Supplement: Supplementary file 1 [file Data_Sheet_1.docx]

Clinical development and marketing application review times for novel orphan-designated drugs

Ebru Demirci^1*^, Jennifer Knicley^1^, Lori Fiorentino^1^

^1^Global Regulatory Affairs, Pharming Group N.V., Leiden, The Netherlands

Supplementary Material

## Supplementary Figures


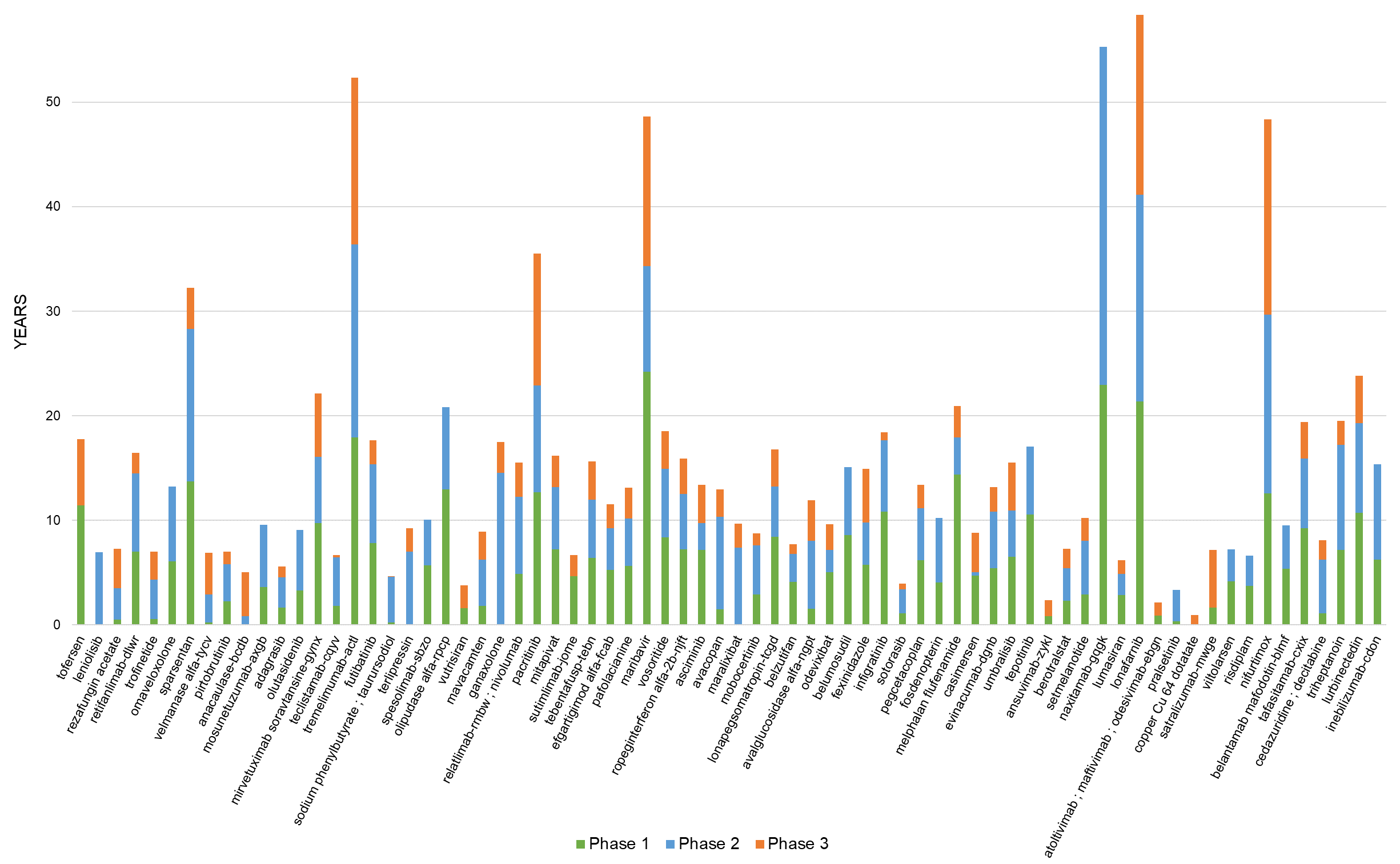


**Supplementary Figure 1.** Clinical development time of each novel orphan-designated drug for each clinical development phase (stacked) Data source: Cortellis Clinical Trials Intelligence.
